# Influence of Pelvic Floor Disorders on Sleep Quality in Women

**DOI:** 10.3390/jpm14030320

**Published:** 2024-03-20

**Authors:** Rocío Adriana Peinado-Molina, Sergio Martínez-Vázquez, Antonio Hernández-Martínez, Juan Miguel Martínez-Galiano

**Affiliations:** 1 Department of Nursing, University of Jaen, 23071 Jaen, Spain; rpmolina@ujaen.es (R.A.P.-M.); svazquez@ujaen.es (S.M.-V.); 2 Department of Nursing, Physiotherapy and Occupational Therapy, University of Castilla-La Mancha, 13071 Ciudad Real, Spain; antonio.hmartinez@uclm.es; 3 Consortium for Biomedical Research in Epidemiology and Public Health (CIBERESP), 28029 Madrid, Spain

**Keywords:** pelvic floor dysfunction, sleep quality, women’s health

## Abstract

Pelvic floor disorders, the impact of their symptoms, and their association with sleep quality and sleep disorders is a little studied area. The aim of this study was to determine if an association exists between pelvic floor disorders in women and sleep disorders. An observational study was conducted among women in Spain during 2021 and 2022. A self-developed questionnaire was used to collect sociodemographic and employment data, previous medical history and health status, lifestyle and habits, obstetric history, and health problems. A validated questionnaire, the Pittsburgh Sleep Quality Index (PSQI), was used to assess the quality of sleep. The presence and impact of pelvic floor problems was assessed with the Pelvic Floor Distress Inventory (PFDI-20). Odds ratios (OR) and adjusted odds ratios (aOR) with their respective 95% confidence intervals were calculated using logistic regression. A total of 1396 women participated in the study. The total PSQI indicated that 75.36% (1052) of women have altered general sleep quality. Women with pelvic floor disorders have a higher probability of developing sleep alterations (aOR: 1.32; 95% CI: 1.22–1.42; for every 20 points). A high BMI (aOR: 1.04; 95% CI: 1.01–1.07; for each point) and the presence of musculoskeletal disorders (aOR: 3.14; 95% CI: 1.20–8.27) are also associated with sleep quality in women. Women with pelvic floor disorders are more likely to develop sleep disorders, probably due to all the discomfort they entail.

## 1. Introduction

Sleep is an active physiological and complex process; its variability in terms of quality and duration has cultural, social, environmental, psychological, behavioral, and pathophysiological influences [1]. Moreover, sleeping is an activity inherent to human nature and has a significant effect on maintaining physical and mental health and a good quality of life [2,3].

Insomnia is a public health problem, affecting up to a third of the population in the USA [4,5] and approximately one-tenth of the Korean population [6]. Only 27–52% of people suffering from insomnia consult with a specialist [7,8]. Recent studies report that sleep disorders are more frequent among women, finding a prevalence of up to 45.5% of women with sleep problems lasting at least one year [3,9,10].

Sleep alterations and sleep disorders have a negative influence on health and weaken the immune system, with direct implications for the risk of infectious and inflammatory diseases [11], as well as causing a greater probability of chronic health conditions, such as diabetes, hypertension, increased cardiovascular risk, cancer, and emotional stress; and psychological problems such as depression and anxiety, substance abuse, and suicidal thoughts, among others [1,12,13,14,15].

Among the risk factors, being a woman has been associated with a greater probability of developing sleep disorders and having worse sleep quality. Additionally, different studies have identified other factors, such as some musculoskeletal issues, osteoporosis, fractures, back problems [16], psychiatric problems such as depression and anxiety [17], and pelvic floor disorders and the impact of their symptoms [18,19,20,21,22,23].

Pelvic floor dysfunction in women encompasses a wide range of clinical disorders: urinary incontinence, pelvic organ prolapse, fecal incontinence, and pelvic–perineal region pain syndrome [24,25,26]. The prevalence of these disorders ranges between 1.9% and 46.50% according to the type of dysfunction and the study population. Moreover, it is a globally relevant problem and is poorly diagnosed [24,25,26].

Studies that have investigated the relationship between pelvic floor disorders and sleep are scarce [18,19,20,21,22,23,27,28,29]. Therefore, this investigation recommends that more studies should be carried out in this area, as not all disorders are addressed. Considering the above, and due to the high prevalence of the problem and the repercussions on the women experiencing them, we aimed to study the association between the different pelvic floor disorders that women may develop and the presence of sexual dysfunctions.

## 2. Methods

### 2.1. Design and Subject Selection

An observational study was carried out among women in Spain during 2021 and 2022. Exclusion criteria included women under 18 years of age, women who had difficulty understanding Spanish, women who had given birth within the previous 12 months, women who were pregnant, and women who had mental health or cognitive disorders that could affect data collection.

Given the lack of previous studies from which the prevalence of sleep alterations could be extracted as a function of pelvic floor dysfunction, we opted to determine the power of contrasts. Thus, accepting an alpha risk of 0.05 in a two-way contrast, with 344 subjects in the group of women without sleep alteration and 1052 in the group of women with sleep alteration, the power of the hypothesis contrast was 100%. To be defined as statistically significant, the difference between the average in the group without sleep alteration must be 32.96 points, and 59.19 points in the group with sleep alteration, according to the Pelvic Floor Distress Inventory scale (PFDI-20) (Pooled Standard Deviation: 49.32).

### 2.2. Information Sources and Study Variables

Participants were recruited extensively by publicly disseminating the research in centers where women engaged in various activities, women’s associations, and neighborhood associations. Additionally, participants from social and educational groups and workshops at healthcare centers were considered. Furthermore, information was spread among the nurse’s patient roster through healthcare centers, encompassing elderly care facilities and day centers, among others. Initially, after recruitment and obtaining informed consent from the women, trained observers conducted the interviews consecutively to obtain data.

Sociodemographic data, work data, personal history, health status, lifestyle and habits, obstetric history, physical activity (International Physical Activity Questionnaire, IPAQ), and health problems were collected using a tailor-made questionnaire, which had been previously piloted. In addition, to assess the quality of sleep, the validated Pittsburgh Sleep Quality Index (PSQI) [30] was used, which consists of 19 self-assessed questions. Based on these questions, seven components were elaborated on, which evaluated different aspects of sleep quality: subjective sleep quality, sleep latency, sleep time, total sleep efficiency, sleep disorders, consumption of hypnotic drugs, and daytime dysfunction. Each aspect had a score that ranged between 0 (no problem) and 3 points (severe problem). Finally, to determine the scale’s total score, the scores of these components are added together, resulting in a minimum score of 0 points and a maximum of 21 points. Participants with a total score of 0 to 4 are considered to have good sleep quality, and scores equal to or greater than 5 are interpreted as poor sleep quality [31].

To assess the presence of pelvic floor problems, the Pelvic Floor Distress Inventory (PFDI-20) was used [32]. The PFDI-20 includes 20 items divided into three symptom scales: symptoms of pelvic organ prolapse (POPDI-6) (questions 1 to 6); colorectal-anal symptoms (CRADI-8) (questions 7–14); and urinary symptoms (UDI-6) (questions 15–20). The UDI-6 subscale contains six items that evaluate urinary symptoms with a maximum score of 100 points, the CRADI-8 subscale evaluates colorectal symptoms with a score of 100 points, and the POPDI-6 subscale contains six items that evaluate prolapse symptoms with a maximum score of 100 points. The PFDI-20 has a total score of 300 points, a higher score indicates a greater burden of symptoms. To establish if a woman has suffered prolapse, an affirmative response was needed for question 3 of the PFDI-20; for fecal incontinence, question 9 or 10 of the PFDI-20; for urinary incontinence, questions 16, 17, or 18 of the PFDI-20; and for pelvic pain, question 20 of the PFDI-20.

### 2.3. Statistical Analysis

The statistical program used for the analysis of the information was SPSS 28.0.

First, descriptive statistics were carried out using absolute and relative frequencies for categorical variables and means with standard deviation (SD) for continuous variables.

Next, a bivariate analysis was performed between the presence of pelvic floor disorders and sleep quality. For this purpose, the Pearson Chi-Square test was used, and the odds ratio (OR) and their respective 95% confidence intervals were calculated.

Finally, a bivariable and multivariable analysis was performed between the different factors and the presence of sleep disorders by means of logistic regression. Crude odds ratios (OR) and adjusted odds ratios (aOR) were estimated for the following factors: age, BMI, alcohol consumption, smoking habit, number of pregnancies, vaginal births, miscarriages, cesarean sections, menopausal status, instrumental birth, perineal trauma (episiotomy and tears), physical activity, and associated pathologies. The level of statistical significance was considered as *p* < 0.05.

### 2.4. Ethical Considerations

This study received a favorable opinion from the Research Ethics Committee of the province of Jaen, reference number SPCV-0220/0302-N-20. Before starting the questionnaire, the women had to read an information sheet about the study and its objectives and confirm their consent to participate.

## 3. Results

A total of 1396 women participated in the study, with a mean age of 44.40 years (SD = 14.70) and a mean BMI of 25.03 (SD = 4.77). In all, 57.4% (802) of the sample were married, and 35% (488) had a median income level of EUR 1000–1999. A total of 14.3% (200) of the women smoked and 54.4% (759) drank occasionally.

Regarding personal and obstetric history, 29% (405) were in post menopause, 33.4% (466) of women had a disease diagnosis, of which 6.6% (92) were musculoskeletal pathologies, 2.7% (37) were respiratory, and 1.8% (25) related to mental health. A total of 78.3% (1093) had been pregnant previously. Regarding the type of delivery, 67.2% (917) had experienced a vaginal birth, and 26.3% (397) an instrumental one. (Table 1).

Table 2 shows the distribution of responses regarding sleep quality according to the Pittsburgh Questionnaire (PSQI), as well as the variables that assess the variety of disorders that affect sleep quality. Notably, 32.2% (n = 450) evaluated their sleep quality as “quite bad”. In addition, 45.2% (631) women slept between 5 and 6 h, and 32.2% (450) women used some type of hypnotic medication once or twice a week. In general, the total PSQI showed that 75.36% (1052) of the women who participated presented an alteration in the quality of sleep at a global level.

Next, as shown in Table 3, the relationship between pelvic floor disorders and sleep disorders was studied, with a statistically significant association observed in all cases (*p* < 0.05). Specifically, women who had a urinary incontinence OR of 1.83 (95% 1.44–2.35), a fecal incontinence OR of 2.13 (95% CI: 1.32–3.44), a pelvic pain OR of 3.45 (95% CI: 2.27–5.25), or a prolapse OR of 1.77 (95% CI: 1.19–2.62) presented a higher probability of sleep disturbances than those who did not have these dysfunctions. In addition, the relationship between the impact of symptoms and the presence of sleep disturbances was analyzed, with statistical associations observed with the four scales (*p* < 0.05). Overall, women with sleep disorders presented an average of 26.33 points (95% CI: 21.65–31.01) more in the impact of pelvic floor symptoms (PFDI-20) than those who did not have sleep disorders.

Finally, a multivariable analysis was carried out, as shown in Table 4. An elevated BMI was identified as a risk factor for having sleep disorders, with an aOR of 1.04 (95% CI: 1.01–1.07). Furthermore, the results indicated that women with musculoskeletal conditions, relative to other conditions, were more likely to have sleep disturbances with an aOR of 3.14 (95% CI: 1.20–8.27). In addition, a greater impact of the symptoms of pelvic floor problems, as assessed by the PFDI-20 scale, increased the probability of having worse sleep quality with an aOR of 1.32 (95% CI: 1.22–1.42; for every 20 points).

## 4. Discussion

Pelvic floor disorders and the impact of their symptoms influence the quality of women’s sleep. Other factors associated with sleep disturbances include BMI and musculoskeletal pathologies.

For the strengths of this study, it should be noted that the study’s sample size is large and, as a novel aspect, included all pelvic floor disorders, which had not previously been included jointly by other authors. On the other hand, for its limitations, the influence of possible selection and memory biases was controlled a priori by using a previously piloted questionnaire that had been prepared in a level of language that was easy to read and understand. To avoid confounding bias, all the variables that could influence the obtained results, such as menopausal status, BMI, age, medical history, among others, were included in the multivariate analysis. Finally, although a clinical evaluation of the women was not carried out through clinical diagnostic means, the instruments used to detect pelvic floor disorders and sleep disorders were questionnaires [30,32], validated in a population similar to ours, and are internationally accepted as instruments to detect possible pelvic floor problems [33,34,35,36,37].

Regarding sleep quality, the results obtained in our study show a high prevalence of sleep alterations, with 75.36% of the participants reporting poor sleep quality. The high prevalence of sleep alterations may be due to the low existing cut-off point. In fact, our results are higher than those identified in other studies [6,38,39,40]. In a cross-sectional descriptive study carried out in Spain [39] with 2144 participants (1173 women and 971 men) with ages ranging from 43 to 71 years, 44.6% of women had poor sleep quality vs. 75.36% in our study, a difference of 30 percentage points. In a cross-sectional descriptive study also carried out in a region of Spain (Community of Madrid), with a sample of 240 participants (165 women and 75 men), the prevalence (40%) was also lower than that obtained in our results [38]. In a cross-sectional descriptive study carried out in 11 Latin American countries [40] in which 6079 women participated, using the same sleep quality identification method and instrument, 46.2% of the participants were detected to have poor sleep quality, a prevalence lower than ours. In another cross-sectional study conducted on a Korean population [6] with 2695 participants between the ages of 19 and 69, including 1350 women, the global prevalence of insomnia symptoms was 10.7%, well below that found in our study.

Our results did not detect an association between age and sleep disturbances, as found in other studies [41,42,43,44] However, it is important to highlight that the distribution of sleep disorders between age groups varies. Thus, in line with the literature, it was mainly found that the older the age, the higher the probability of having worse sleep quality. For example, in a multicenter cohort study [45] conducted in Chicago surveying 814 men and women, age was associated with poor sleep quality. On the other hand, Uhlig et al. [46], opposite to our results, found sleep alterations in those over 50 years of age in their cross-sectional descriptive study carried out in Norway with 93,860 participants older than 20 years (22,728 women).

BMI, in line with our results, has been related to sleep quality in a recent cross-sectional study conducted in South Korea in 2016 [47] with 1165 participants (737 men and 428 women) aged between 19 and 64 years; the study identified that a high BMI was associated with poor sleep quality. Other authors also found this association [21,46].

On the other hand, different authors [20,48] have established an association between menopausal state and insomnia and poorer sleep quality, which could not be documented in our results.

Mental health is a factor that influences the sleep of women [49], an association not observed in our results. However, an association between musculoskeletal pathologies and sleep disturbances was observed in our study, in agreement with that observed in other studies [46,50].

Finally, in line with some researchers [18,19,20,21,22,23,27,28,29], pelvic floor disorders were found to influence sleep quality. Specifically, regarding urinary incontinence, the authors Yilmaz Bulut and Altay and Winkelman et al. found an association in cross-sectional studies with 140 women carried out in Turkey and with 640 women carried out in San Francisco, respectively [22,23]. Concerning uterine prolapse, Humalajärvi et al., in their prospective study [19] in Finland involving 322 women, found that the women who had prolapse scored significantly worse in the sleep dimensions. Regarding pelvic pain, and in line with our results, in a case-control study conducted in Turkey [29] with 157 women using the same sleep quality scale as used in the present study, it was observed that women with chronic pelvic pain had poorer sleep quality, an association also shown by other authors [28]. Luo Y et al. [27] in a multicenter cross-sectional study carried out in China with 1250 participants, observed that sleep disorders were related to double incontinence, that is, urinary and fecal; however, this association was not found when the woman only suffered from fecal incontinence, contrary to the results found in our investigation.

Each stage in a woman’s life increases the risk of suffering from sleep disorders and requires different management strategies that must be implemented considering the circumstances of each moment and state in which the woman finds herself. The results show that women at any age are one of the most vulnerable groups to suffer from the public health problem of sleep disorders. Efforts are currently being made to mitigate many of the risk factors associated with sleep disturbances. For this reason, strategies must be applied that pay attention to new factors that influence sleep quality and disorders, such as pelvic floor disorders, both in preventing their appearance and in addressing them once they are already present. There is growing concern in public health regarding the importance of sleep. This phenomenon has increased due to social demand for strategies aimed at improving sleep quality in the population. However, there is no standardized consensus on how to apply these strategies.

## 5. Conclusions

In conclusion, women with pelvic floor disorders have a higher probability of developing sleep alterations. A high BMI and musculoskeletal disorders also negatively influence the quality of sleep.

## Figures and Tables

**Table 1:** Sociodemographic and clinical characteristics of the study sample.

Variable	n (%)	Mean (SD)
**Age**		44.40 (14.70)
<30 years	212 (15.4)	
30–49.9 years	768 (55.0)	
≥50 years	416 (29.8)	
**BMI**		25.03 (4.77)
Normal weight < 25	797 (57.1)	
Overweight 25–29.9	395 (28.3)	
Obesity ≥ 30	204 (14.6)	
**Civil status**		
Single	317 (22.7)	
Separated	18 (1.3)	
Divorced	72 (5.2)	
Widowed	65 (4.7)	
Common-law couple	122 (8.7)	
Married	802 (57.4)	
**Education level**		
Primary level, uncompleted	69 (4.9)	
Primary level, completed	90 (6.4)	
Secondary level	92 (6.6)	
Baccalaureate	177 (12.7)	
University level	968 (69.3)	
**Income level**		
EUR < 1000	192 (13.8)	
EUR 1000–1999	488 (35.0)	
EUR 2000–2999	415 (29.7)	
EUR > 3000	301 (21.6)	
**Alcohol consumption**		
Never	341 (24.4)	
Occasionally	759 (54.4)	
Only weekends	138 (9.9)	
Frequently	135 (9.7)	
Daily	23 (1.6)	
**Smoking habit**	200 (14.3)	
**Pregnancy**		
None	303 (21.7)	
One	183 (13.1)	
Two or more	910 (65.2)	
**Miscarriages**		
None	1012 (72.5)	
One	277 (19.8)	
Two or more	107 (7.7)	
**Cesarean section**		
None	1142 (81.8)	
One	183 (13.1)	
Two or more	71 (5.1)	
**Vaginal birth**		
None	455 (32.6)	
One	278 (19.9)	
Two or more	663 (47.5)	
**Instrumental birth**	397 (26.3)	
**Menopausal status**	405 (29.0)	
**Urinary incontinence**	779 (55.8)	
**Fecal incontinence**	149 (10.7)	
**Prolapse**	199 (14.3)	
**Pelvic pain**	266 (19.1)	
**Illness**	466 (33.4)	
**Cardiovascular disorder**	120 (8.6)	
**Respiratory disorder**	37 (2.7)	
**Endocrine disorder**	146 (10.5)	
**Gynecological disorder**	40 (2.9)	
**Musculoskeletal disorder**	92 (6.6)	
**Neurological disorder**	34 (2.4)	
**Neoplastic disease**	11 (0.8)	
**Gastrointestinal disorder**	41 (2.9)	
**Dermatological disorder**	22 (1.6)	
**Mental health disorder**	25 (1.8)	
**Nephro-urological disorder**	10 (0.7)	
**Immunological disorder**	10 (0.7)	
**Ophthalmology-ENT disorder**	23 (1.6)	

**Table 2:** Response distribution for sleep quality.

Variable	Evaluation According to the Pittsburgh Questionnaire (PSQI)			
	**Very Good** **% (n)**	**Quite Good** **% (n)**	**Poor** **% (n)**	**Very Poor** **% (n)**
**Subjective sleep quality**	10.7 (150)	53.6 (748)	32.2 (450)	3.4 (48)
	**≤15 min** **% (n)**	**16–30 min** **% (n)**	**31–60 min** **% (n)**	**60 min** **% (n)**
2. **Sleep latency**	22.3 (311)	38.8 (541)	22.3 (311)	16.7 (233)
	**More than 7 h % (n)**	**Between 6 and 7 h** **% (n)**	**Between 5 and 6 h** **% (n)**	**Less than 5 h % (n)**
3. **Sleep duration**	17.8 (249)	29.5 (412)	45.2 (631)	7.4 (104)
	**>85%** **% (n)**	**75–84%** **% (n)**	**65–74%** **% (n)**	**<65%** **% (n)**
4. **Sleep habit efficiency**	42.4 (592)	23.8 (332)	16.0 (224)	17.8 (248)
	**Never in the last month % (n)**	**Less than once a week % (n)**	**One or two times a week % (n)**	**Three or more times a week % (n)**
5. **Sleep disturbances**	6.2 (86)	75.6 (1056)	17.6 (245)	0.6 (9)
6. **Use of sedative-hypnotics**	10.7 (150)	53.6 (748)	32.2 (450)	3.4 (48)
7. **Daytime dysfunction**	35.1 (490)	38.9 (543)	21.6 (302)	4.4 (61)

**Table 3:** Bivariate analysis of pelvic floor problems and sleep quality.

	Dichotomy of Sleep Quality		Bivariate Analysis	
	No Alteration % (n) n = 344	With Alteration % (n) n = 1052	OR (95% CI)	*p* Value
**Urinary incontinence**				**<0.001**
**No**	31 (191)	69 (426)	**1**	
**Yes**	19.6 (153)	80.4 (626)	**1.83 (1.44–2.35)**	
**Fecal incontinence**				**0.002**
**No**	25.9 (323)	74.1 (924)	**1**	
**Yes**	14.1 (21)	85.9 (128)	**2.13 (1.32–3.44)**	
**Pelvic pain**				**<0.001**
**No**	28.1 (317)	71.9 (813)	**1**	
**Yes**	10.2 (27)	89.8 (239)	**3.45 (2.27–5.25)**	
**Prolapse**				**0.004**
**No**	26 (311)	74 (886)	**1**	
**Yes**	16.6 (33)	83.4 (166)	**1.77 (1.19–2.62)**	
**Score impact of pelvic floor problems**	**No** **Mean (SD)**	**Yes** **Mean (SD)**	**Mean difference (95% CI)**	***p***
**Prolapse symptoms**	8.82 (11.56)	16.40 (17.73)	**7.59 (5.57–9.58)**	**<0.001**
**Colorectal-Anal symptoms**	10.27 (12.45)	17.73 (17.88)	**7.47 (5.75–9.17)**	**<0.001**
**Urinary symptoms**	13.77 (15.59)	25.06 (24.30)	**11.29 (9.08–13.49)**	**<0.001**
**Pelvic function disorders Total (PFDI-20)**	32.86 (32.73)	59.19 (52.04)	**26.33 (21.65–31.01)**	**<0.001**

**Table 4:** Bivariate and multivariate analysis sleep quality and associated factors.

	Dichotomy of Sleep Quality		Bivariate Analysis		Multivariate Analysis	
Pelvic Floor Problems	No Alteration n = 344	With Alteration n = 1052	Odds Ratio (95% CI)	*p* Value	Odds Ratio * (95% CI)	*p* Value
**Age n (%)**				0.120		0.131
<30 years	52 (24.5)	160 (75.5)	1		1	
30–49.9 years	204 (26.6)	564 (73.4)	0.90 (0.63–1.28)	0.551	0.60 (0.36–1.01)	0.057
≥50 years	88 (21.2)	328 (78.8)	1.21 (0.82, 1.79)	0.337	0.74 (0.35–1.60)	0.448
**BMI (mean; SD)**	24.11 (4.47)	25.34 (4.82)	**1.06 (1.03–1.09)**	**<0.001**	**1.04 (1.01–1.07)**	**0.028**
**Alcohol consumption n (%)**				0.450		0.917
Never	21.1 (72)	78.9 (269)	1		1	
Occasionally	25.8 (196)	74.2 (563)	0.77 (0.57–1.04)	0.093	0.94 (0.67–1.31)	0.703
Only weekends	26.8 (37)	73.2 (101)	0.73 (0.46–1.16)	0.179	0.97 (0.59–1.58)	0.890
Frequently	23.7 (32)	76.3 (103)	0.86 (0.54–1.38)	0.538	1.06 (0.64–1.76)	0.812
Daily	30.4 (7)	69.6 (16)	0.61 (0.24–1.54)	0.298	0.66 (0.24–1.82)	0.427
**Smoking habit n (%)**				0.510		0.698
No	24.3 (291)	75.7 (905)	1		1	
Yes	26.5 (53)	73.5 (147)	0.89 (0.63–1.25)		0.93 (0.65–1.34)	
**Pregnancy n (%)**				0.260		0.847
None	27.7 (84)	72.3 (219)	1		1	
One	39 (21.3)	78.7 (144)	1.42 (0.92–2.19)		1.12 (0.53–2.34)	0.765
Two or more	24.3 (221)	75.7 (689)	1.20 (0.90–1.61)		0.96 (0.38–2.41)	0.924
**Miscarriages n (%)**				0.648		0.937
None	25.3 (256)	74.7 (756)	1		1	
One	23.1 (64)	76.9 (213)	1.13 (0.82–1.54)	0.455	1.04 (0.72–1.49)	0.841
Two or more	22.4 (24)	77.6 (83)	1.17 (0.73–1.89)	0.515	1.10 (0.64–1.89)	0.734
**Cesarean section n (%)**				**0.043**		0.163
None	26 (297)	74 (845)	**1**		1	
One	18.6 (34)	81.4 (149)	**1.54 (1.04–2.29)**		1.70 (0.98–2.92)	0.059
Two or more	18.3 (13)	81.7 (58)	1.57 (0.85–2.90)		1.78 (0.67–4.74)	0.247
**Vaginal birth n (%)**				0.200		0.629
None	25.5 (116)	74.5 (339)	1		1	
One	20.5 (57)	79.5 (221)	1.33 (0.93–1.90)		1.36 (0.67–2.73)	0.395
Two or more	25.8 (171)	74.2 (492)	0.99 (0.75–1.30)		1.21 (0.51–2.89)	0.668
**Instrumental birth n (%)**				**0.014**		0.053
No	26.3 (271)	73.7 (758)	1		1	
Yes	19.9 (73)	80.1 (294)	**1.44 (1.08–1.93)**		1.42 (0.00–2.02)	
**Menopausal status n (%)**				0.139		0.449
No	25.7 (255)	74.3 (736)	1		1	
Yes	22 (89)	78 (316)	1.23 (0.93–1.62)		0.80 (0.45–1.42)	
**Episiotomy n (%)**				0.914		0.122
No	24.8 (181)	75.2 (550)	1		1	
Yes	24.5 (163)	75.5 (502)	1.01 (0.79–1.29)		0.74 (0.50–1.09)	
**Tear n (%)**				0.330		0.892
No	25.5 (222)	74.5 (648)	1		1	
Yes	23.2 (122)	76.8 (404)	1.13 (0.88–1.46)		1.02 (0.74–1.42)	
**Physical activity (IPAQ) n (%)**				**0.054**		0.519
Low	71 (19.9)	285 (80.1)	1		1	
Medium	197 (26.6)	543 (73.4)	**0.69 (0.51–0.93)**	**0.016**	0.83 (0.60–1.15)	0.256
High	76 (25.3)	224 (74.7)	0.74 (0.51–1.06)	0.100	0.90 (0.61–1.33)	0.581
**Cardiovascular disorder n (%)**				**0.001**		0.565
No	25.8 (329)	74.2 (947)	1		1	
Yes	12.5 (15)	87.5 (105)	**2.43 (1.40–4.24)**		1.21 (0.64–2.28)	
**Pulmonary disorder n (%)**				0.230		0.701
No	24.9 (338)	75.1 (1021)	1		1	
Yes	16.2 (6)	83.8 (31)	1.71 (0.71–4.14)		1.20 (0.46–3.02)	
**Endocrine disorder n (%)**				0.225		0.937
No	25.1 (314)	74.9 (936)	1		1	
Yes	20.5 (30)	79.5 (116)	1.30 (0.85–1.98)		0.94 (0.59–1.48)	
**Gynecological disorder n (%)**				0.151		0.380
No	24.9 (338)	75.1 (1018)	1		1	
Yes	15 (6)	85 (34)	1.88 (0.78–4.52)		1.52 (0.60–3.86)	
**Musculoskeletal Disorder n (%)**				**<0.001**		**0.019**
No	26.0 (339)	74.0 (965)	**1**		**1**	
Yes	5.4 (5)	94.6 (87)	**6.11 (2.46–15.18)**		**3.14 (1.20–8.27)**	
**Neurological disorder n (%)**				0.078		0.543
No	25.0 (340)	75.0 (1022)	1		1	
Yes	11.8 (4)	88.2 (30)	2.50 (0.87–7.13)		1.44 (0.45–4.60)	
**Mental health disease n (%)**				0.839		0.343
No	341 (24.6)	75.4 (1044)	1		1	
Yes	3 (27.3)	72.7 (8)	0.87 (0.23–3.30)		0548 (0.10–2.17)	
**Gastrointestinal disorder n (%)**				**0.009**		0.152
No	25.2 (341)	74.8 (1014)	**1**		1	
Yes	7.3 (3)	92.7 (38)	**4.30 (1.31–13.89)**		2.46 (0.72–8.39)	
**Dermatological disorder n (%)**				0.478		0.474
No	24.7 (340)	75.3 (1034)	1		1	
Yes	18.2 (4)	81.8 (18)	1.48 (0.50–4.40)		1.5 (0.44–4.41)	
**Mental health Disorder n (%)**				**0.016**		0.225
No	25 (343)	75 (1028)	**1**		1	
Yes	4 (1)	96 (24)	**8.01 (1.08–59.41)**		3.59 (0.46–28.29)	
**Nephro-urological Disorder n (%)**				0.281		0.837
No	24.7 (343)	75.3 (1043)	1		1	
Yes	10.0 (1)	90.0 (9)	2.96 (0.37–23.45)		0.79 (0.09–7.35)	
**Immunological disorder n (%)**				0.732		0.902
No	24.7 (342)	75.3 (1044)	1		1	
Yes	20.0 (2)	80 (8)	1.31 (0.28–6.20)		1.11 (0.22–5.67)	
**PFDI-20 questionnaire (for every 20 points) (Mean; SD)**	32.86 (32.73)	59.19 (52.04)	**1.35 (1.26–1.45)**	**<0.001**	**1.32 (1.22–1.42)**	**<0.001**

* Adjusted odds ratios (aOR) for the following factors: age, BMI, alcohol consumption, smoking habit, number of pregnancies, vaginal births, miscarriages, cesarean sections, menopausal status, instrumental birth, perineal trauma (episiotomy and tears), physical activity, and associated pathologies.

## Data Availability

The data that support the findings of this study are available from the corresponding author upon reasonable request.
